# Supplementary material for: NFIB facilitates replication licensing by acting as a genome organizer
Source: Nat Commun. 2023 Aug 21;14:5076. doi: 10.1038/s41467-023-40846-1 (PMC10442334; doi:10.1038/s41467-023-40846-1)

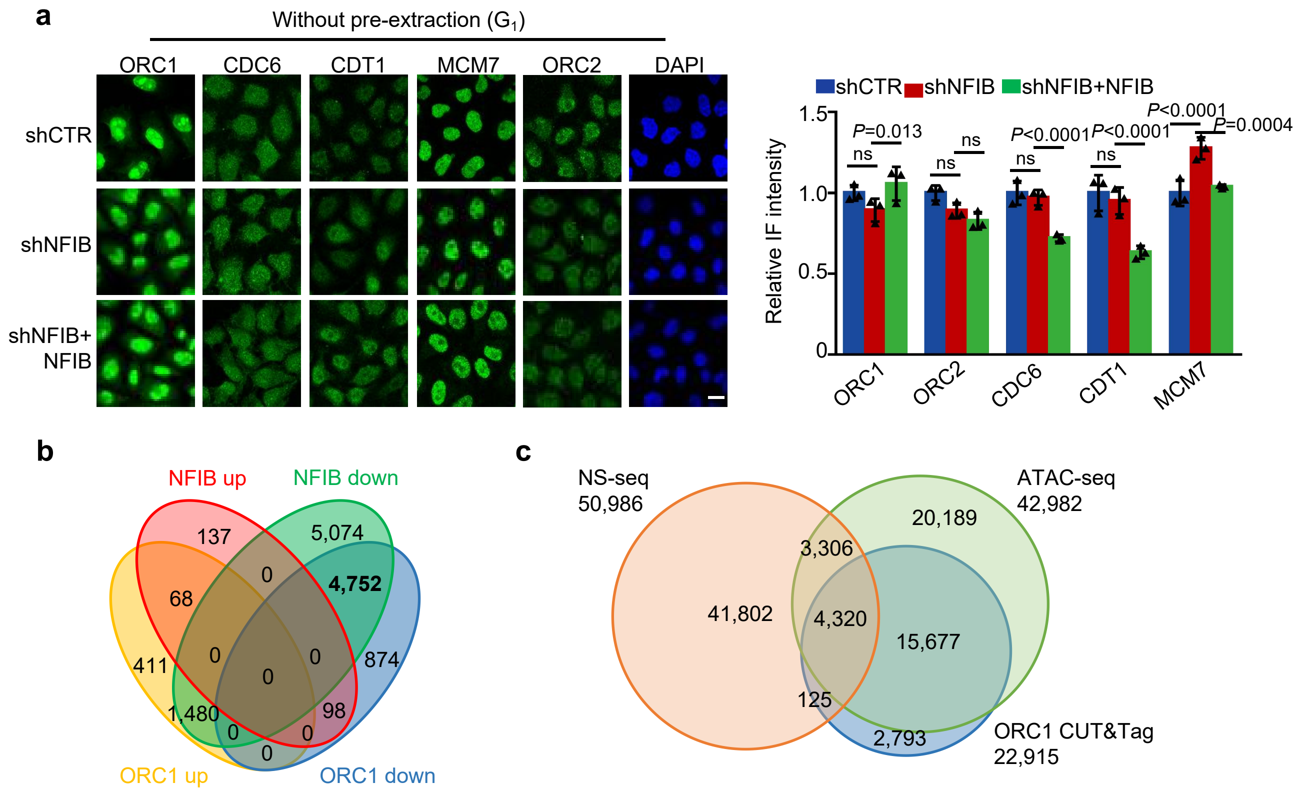

Supplementary Fig. 1 Additional evidence of NFIB-affected pre-RC chromatin binding and chromatin opening, related to Fig. 2 and Fig. 3. **a** U2OS cells were infected with lentiviruses carrying control shRNA, NFIB shRNA and/or FLAG-NFIB and synchronized at G_1 phase for immunofluorescent staining (IF) using the indicated antibodies without pre-extraction. DAPI staining was included to visualize the nucleus (blue). Scale bar, 10 μm. Representative images from triplicate experiments are shown. The relative IF intensity was quantified by ZEN software. Data were presented as mean ± SD of ten sections from each slice for triplicate experiments. *P* values were determined by two-way ANOVA followed by Tukey test. NS, not significant. **b** Venn diagrams showing overlap among altered peaks of NFIB and ORC1 upon NFIB depletion in U2OS cells. Up: signal increased in shNFIB cells. Down: signal decreased in shNFIB cells. **c** The Venn diagrams showing the overlapping regions of ORC1 binding identified by CUT&Tag with NS-seq or ATAC-seq in wild-type U2OS cells.

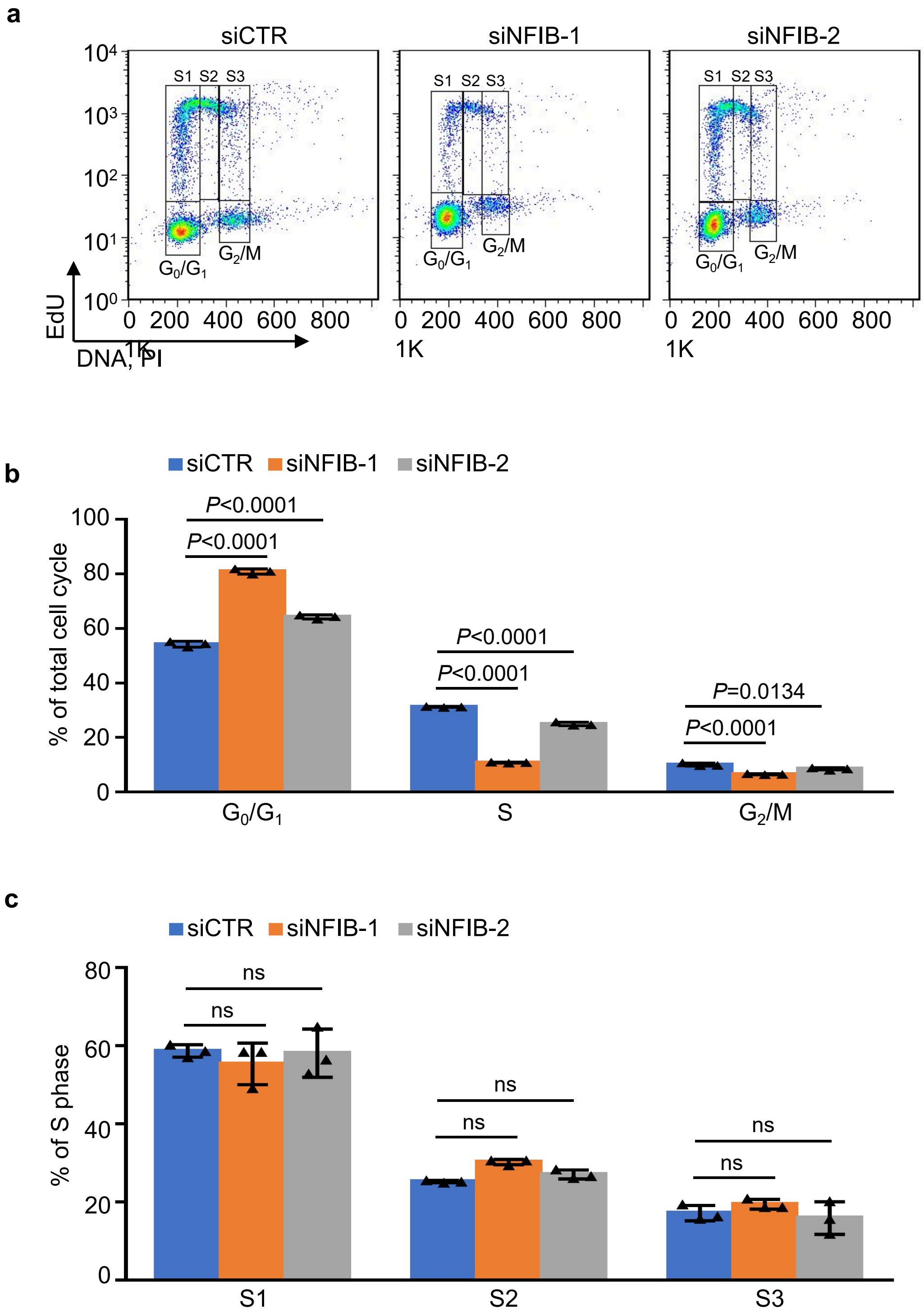

Supplementary Fig. 2 Depletion of NFIB does not change the proportion of cells in early vs middle or late S phase, related to Fig. 3. a FACS analysis of EdU incorporation and DNA content after 1-h pulse with EdU in siCTR or siNFIB U2OS cells. S phase was divided into early-S1, mid-S2, and late-S3 as indicated. **b-c** Summary of the above results showing cell cycle distribution in different phases (G_0/G_1, S, and G_2/M) or sub-S phases (S1, S2, and S3), respectively. The data are presented as the means ± SD for triplicate experiments. *P* values were determined by two-way ANOVA followed by Dunnett test. NS, not significant.

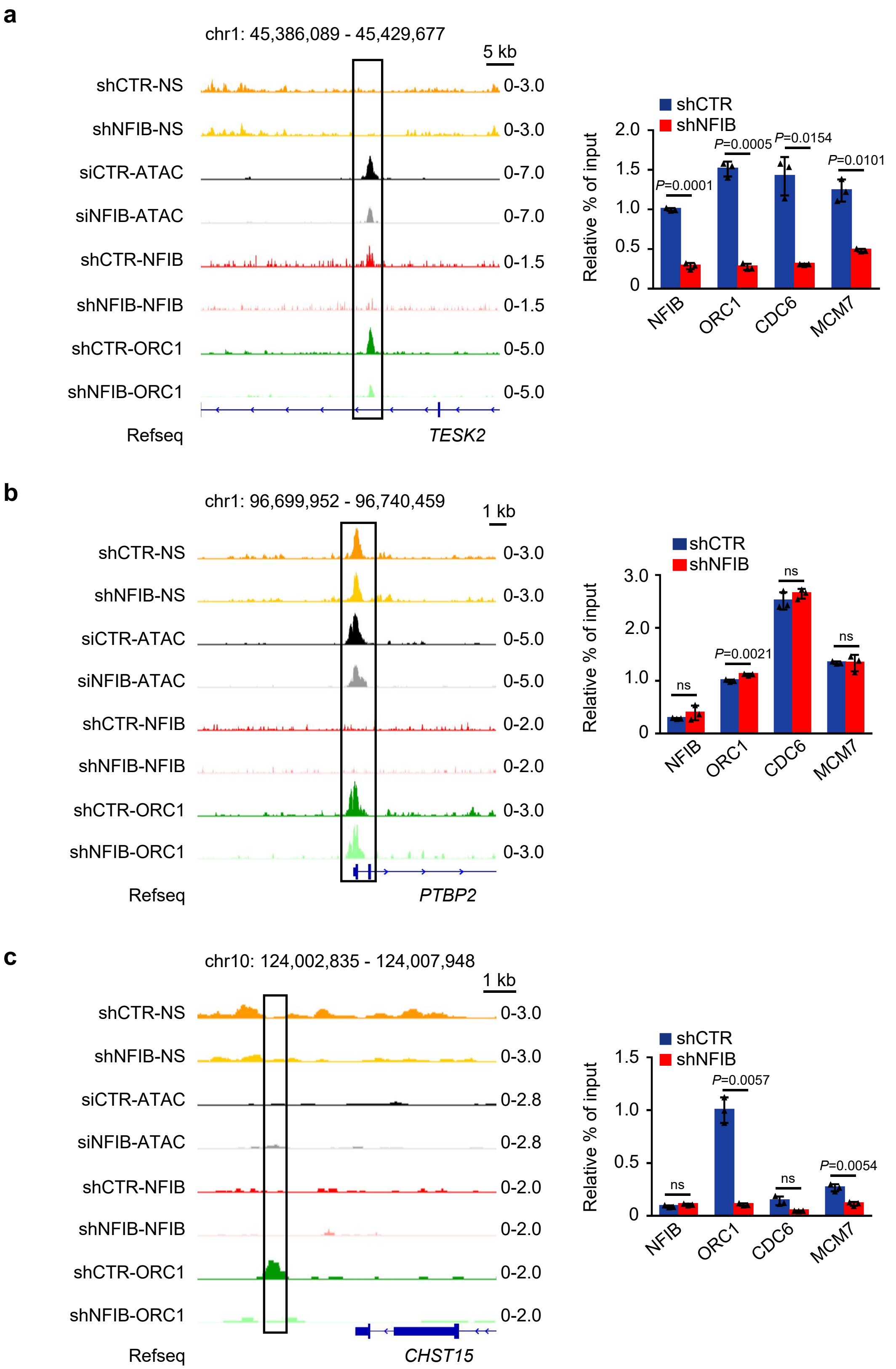

Supplementary Fig. 3. The effect of NFIB depletion on ORC1 binding at dormant origins or non-origins, related to Fig. 4. a-c Tracks as in visualizing representative origins unrelated to NFIB/ORC1 (left). The right panel shows the qChIP results performed in control or shNFIB U2OS cells using antibodies against the indicated proteins (right). The data are presented as the means ± SD for three independent experiments. *P* values were determined by unpaired two-tailed T test. NS, not significant. (**a**) A control site lack of nascent strand signal. (**b**) A control site lack of NFIB binding. (**c**) A control site lack of NFIB binding and nascent strand signal. *TESK2, PTBP2* , and *CHST15* are the Refseq gene names.

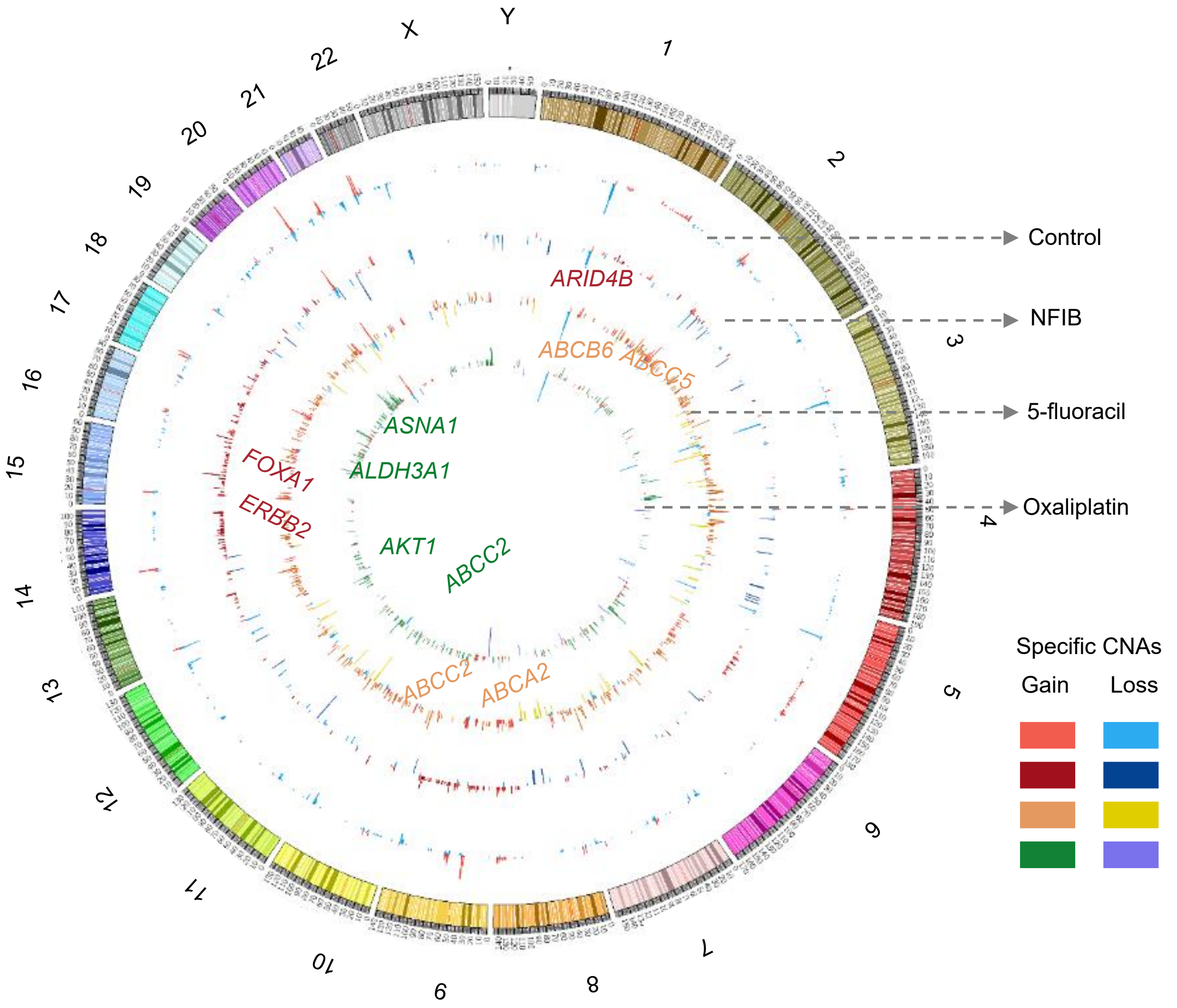

Supplementary Fig. 4 Circos plotting of CNAs detected by genome sequencing of different MCF-10A clones, related to Fig. 7. Circos plotting of CNAs detected by genome sequencing of the indicated cells. Known resistance genes linked to the amplified CNAs identified from NFIB-overexpressing/drug-resistant cells are labeled.

Supplementary Fig. 5

a

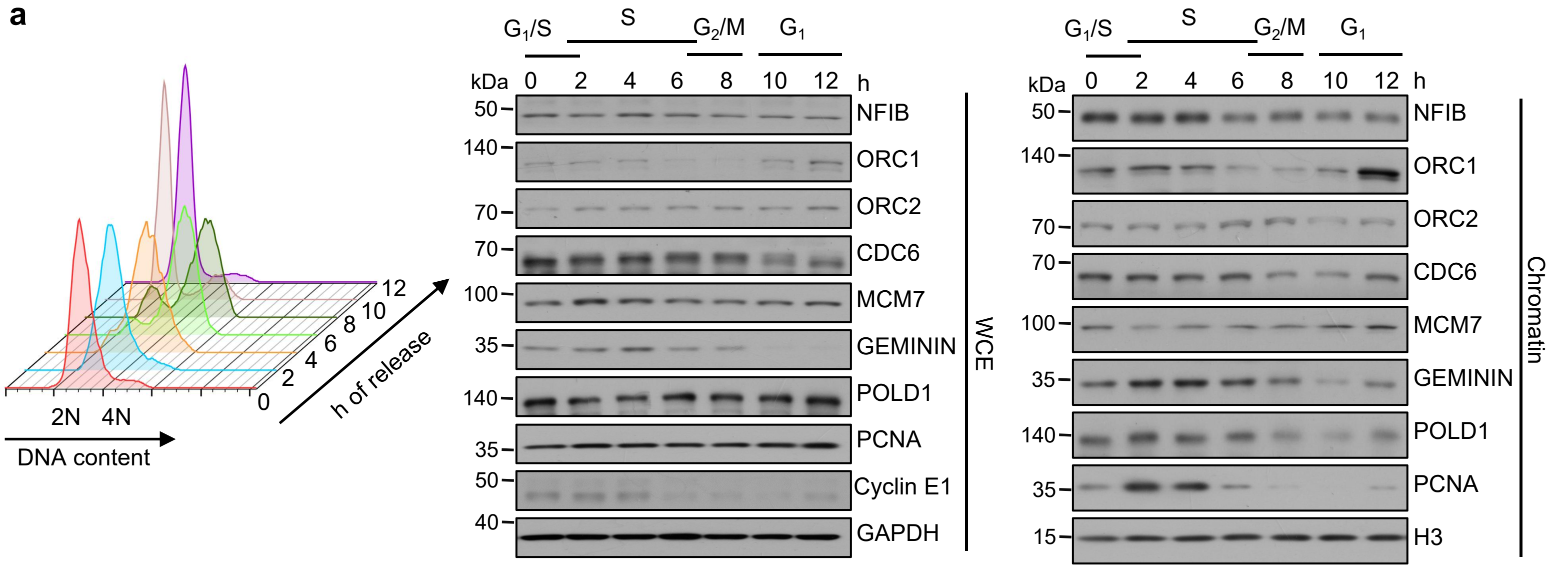

b

BT-474

Drug name	IC_{50} (μM)		*P* value ($lgIC_{50}$)
	siCTR	siNFIB	
Oxaliplatin	5.89e-1	1.44e-1	< 1e-4
Nocodazole	9.67e-1	4.10e-2	< 1e-4
5-fluoracil	4.98e-1	7.44e-2	< 1e-4
Epirubicin HCl	2.06e-2	2.71e-3	< 1e-4

ZR-75-1

Drug name	IC_{50} (μM)		*P* value ($lgIC_{50}$)
	siCTR	siNFIB	
Oxaliplatin	1.18	9.34e-2	< 1e-4
Nocodazole	3.273	1.389	< 1e-4
5-fluoracil	5.96e-1	1.78e-1	< 1e-4
Epirubicin HCl	7.61e-2	4.19e-2	< 1e-4

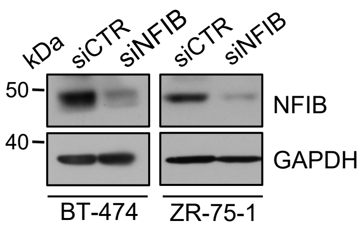

Supplementary Fig. 5 Replication-related biochemical and cellular function of NFIB in breast cancer cells, related to Fig. 8. a BT-474 cells were synchronized by double-thymidine block and released for different hours to allow cells entering different phase of cell cycle as indicated. Whole cell extracts (WCE, left) or chromatin fraction (Chromatin, right) were prepared and subjected to western blotting with antibodies against the indicated proteins. GAPDH or H3 was used as loading control as indicated. The experiment was performed three times with similar observations. **b** Control or NFIB-depleted BT-474 or ZR-75-1 cells were treated with the indicated antineoplastic compounds for 3 days. The growth of cells was measured with the CCK-8 assay. The data are presented as the means ± SD for three independent experiments. *P* values of $lgIC_{50}$ were calculated based on extra sum-of-squares F-test. The efficiency of knockdown was verified by western blotting (right).

a

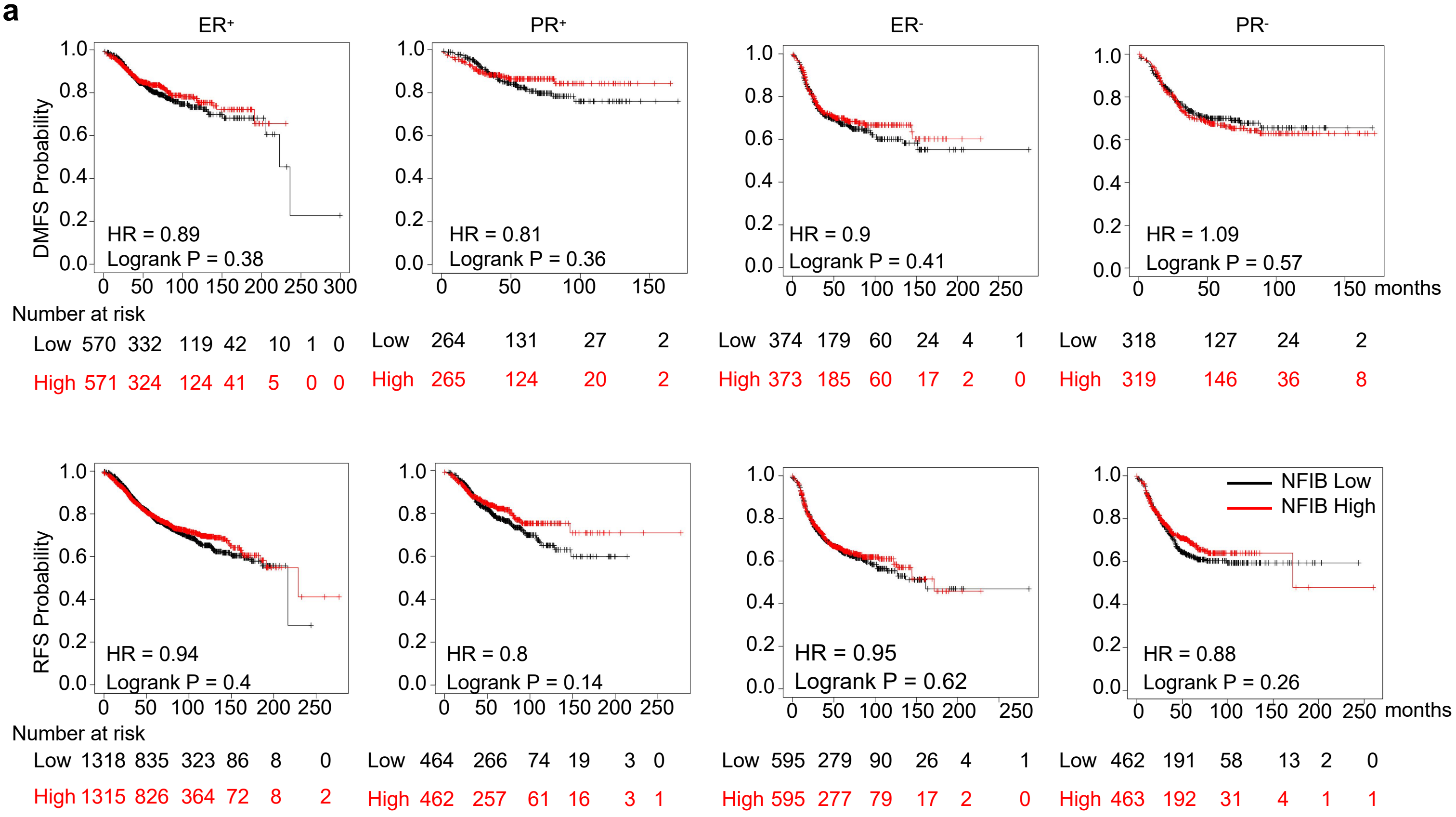

b

Multivariate COX Analysis of DMFS

Factors	ER+ (n = 1141)		ER- (n = 747)		PR+ (n = 529)		PR- (n = 637)	
	P-Value	HR 95% CI	*P*-Value	HR 95% CI	*P*-Value	HR 95% CI	*P*-Value	HR 95% CI
NFIB	0.30	0.87 0.66-1.14	0.36	0.88 0.68-1.15	0.32	0.79 0.50-1.26	0.47	1.11 0.83-1.49
Ki67	1.7e-03	1.59 1.19-2.13	0.56	0.92 0.71-1.20	0.22	1.34 0.84-2.15	0.18	1.22 0.91-1.63

Multivariate COX Analysis of RFS

Factors	ER+ (n = 2632)		ER- (n = 1190)		PR+ (n = 962)		PR- (n = 925)	
	P-Value	HR 95% CI	*P*-Value	HR 95% CI	*P*-Value	HR 95% CI	*P*-Value	HR 95% CI
NFIB	0.41	0.94 0.81-1.09	0.61	0.95 0.78-1.15	0.11	0.79 0.59-1.05	0.24	0.87 0.69-1.10
Ki67	1e-04	1.4 1.19-1.64	0.31	0.9 0.74-1.10	8.1e-03	1.5 1.11-2.03	0.56	1.07 0.85-1.36

Supplementary Fig. 6 Correlation analysis of NFIB expression and prognosis of ER+, PR+ or ER-, PR- subtypes of breast cancer, related to Fig. 8. a Kaplan-Meier survival analysis for the correlation of NFIB expression with distant metastasis-free survival (DMFS) and relapse-free survival (RFS) in ER+, PR+ or ER-, PR- subtypes of breast cancer. **b** Multivariable analyses of DMFS and RFS were performed in ER+, PR+ or ER-, PR- subtypes of breast cancer.

Supplementary Table 1. Mass spectrometry analysis of NFIB-containing protein complex.

Band	Identified proteins	Peptides
226.4 kDa	MYH9	IAQLEEQLDNETK
		QLEEAEEEAQR
		IAQLEEQLDNETKER
		KEEELQAALAR
		TDLLLEPYNK
		QAQQERDELADEIANSSGK
157.8 kDa	LRPPRC	TVLDQQQTPSR
		MEEANIQPNR
		GFTLNDAANSR
		LIASYcNVGDIEGASK
		IQEENVIPR
		VIEEQLEPAVEK
		LDSSAVLDTGK
119.8 kDa	SPT16	DLGFNGAPYR
		INFYcPGSALGR
		NEGNIFPNPEATFVK
96.5 kDa	MCM4	AGIIcQLNAR
92.8 kDa	MCM6	VSGVDGYETEGIR
		IQETQAELPR
		LVFLACCVAPTNPR
90.9 kDa	MCM3	cSVLAAANPVYGR
		LIVNVNDLR
		YVLcTAPR
		DAQPSFSAEDIAK
		TAIHEVMEQGR
		ELISDNQYR
81 kDa	SSRP1	ASSGLLYPLER
77.5 kDa	MCM5	VLGIQVDTDGSGR
		AIAcLLFGGSR
		IPGIIIAASAVR
		QLEAIVR
68.1 kDa	RPA1	DSLVDIIGIcK
		VVPIASLTPYQSK
		VIDQQNGLYR
		VKVETYNDESR
		VIDQQnGLYR
		LFSLELVDESGEIR
		SGGVGGSNTNWK
		DKNEQAFEEVFQNANFR
62.6 kDa	NFIB	KPEKPLFSSASPQDSSPR
		KHPccVLSNPDQK
		AVKDELLSEKPEIK
		HPccVLSNPDQK
		NYVPSYDPSSPQTSQPNGSGQVVGK
		NYVPSYDPSSPQTSQPnGSGQVVGK
		SGVFNVSELVR
		NPPGYLEDSFVK
		EDFVLTVTGK
		NYVPSYDPSSPqTSQPnGSGQVVGK
		DELLSEKPEIK
		YPPHLNPQDTLK
		GIPLESTDGER
		AIAYTWFNLQAR
60.4 kDa	CDT1	GVSqDLLER
55.6 kDa	NFIC	KAPGcVLSNPDQK
		AVKDELLGEKPEVK
		APGcVLSNPDQK
		EDFVLSITGK
		GIPLESTDGER
		TLPSTSSSGSK
44.6 kDa	NFIX	GIPLESTDGER
		SITSPPSTSTTK

FACS information for Fig.1g

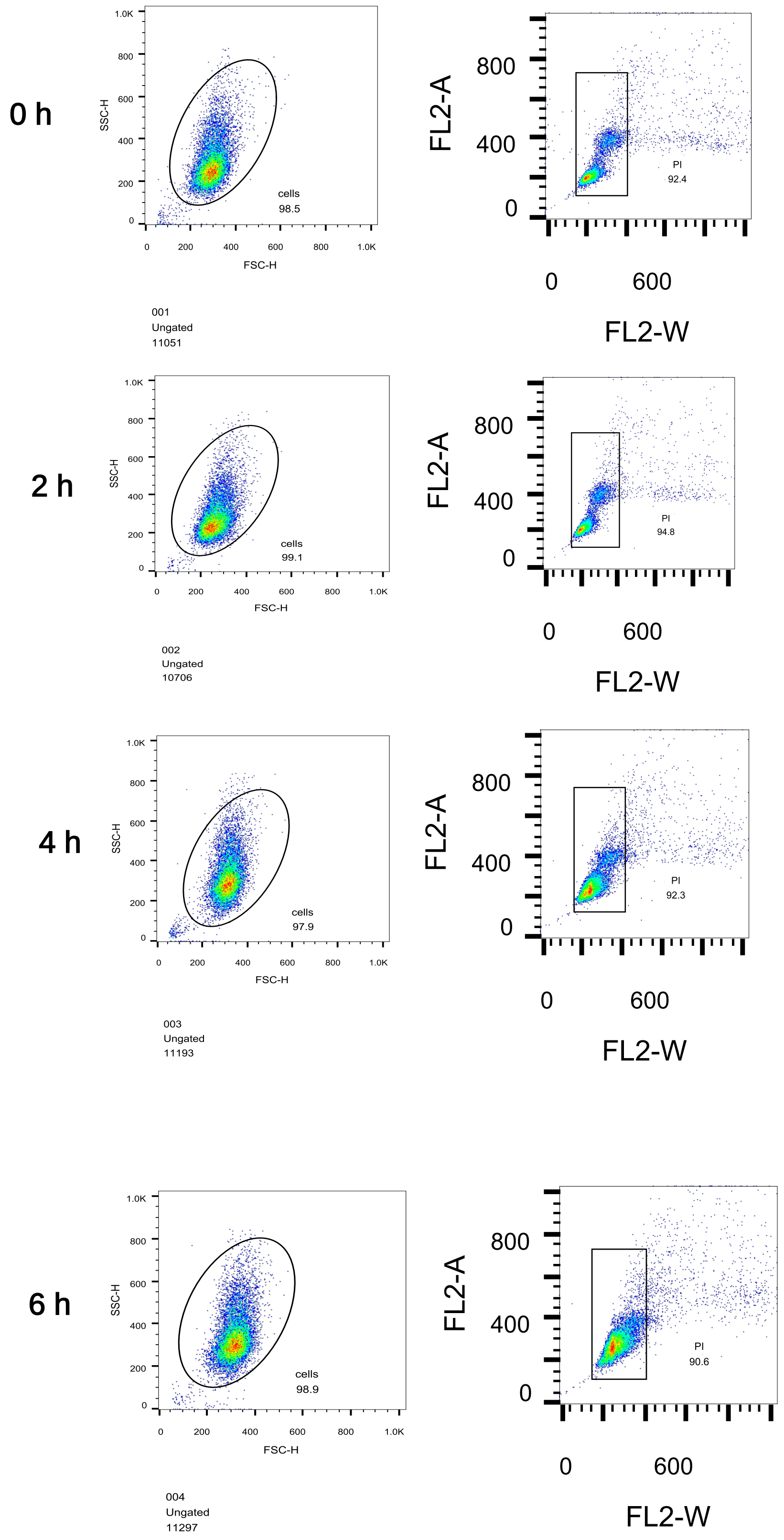

FACS information for Fig.1g

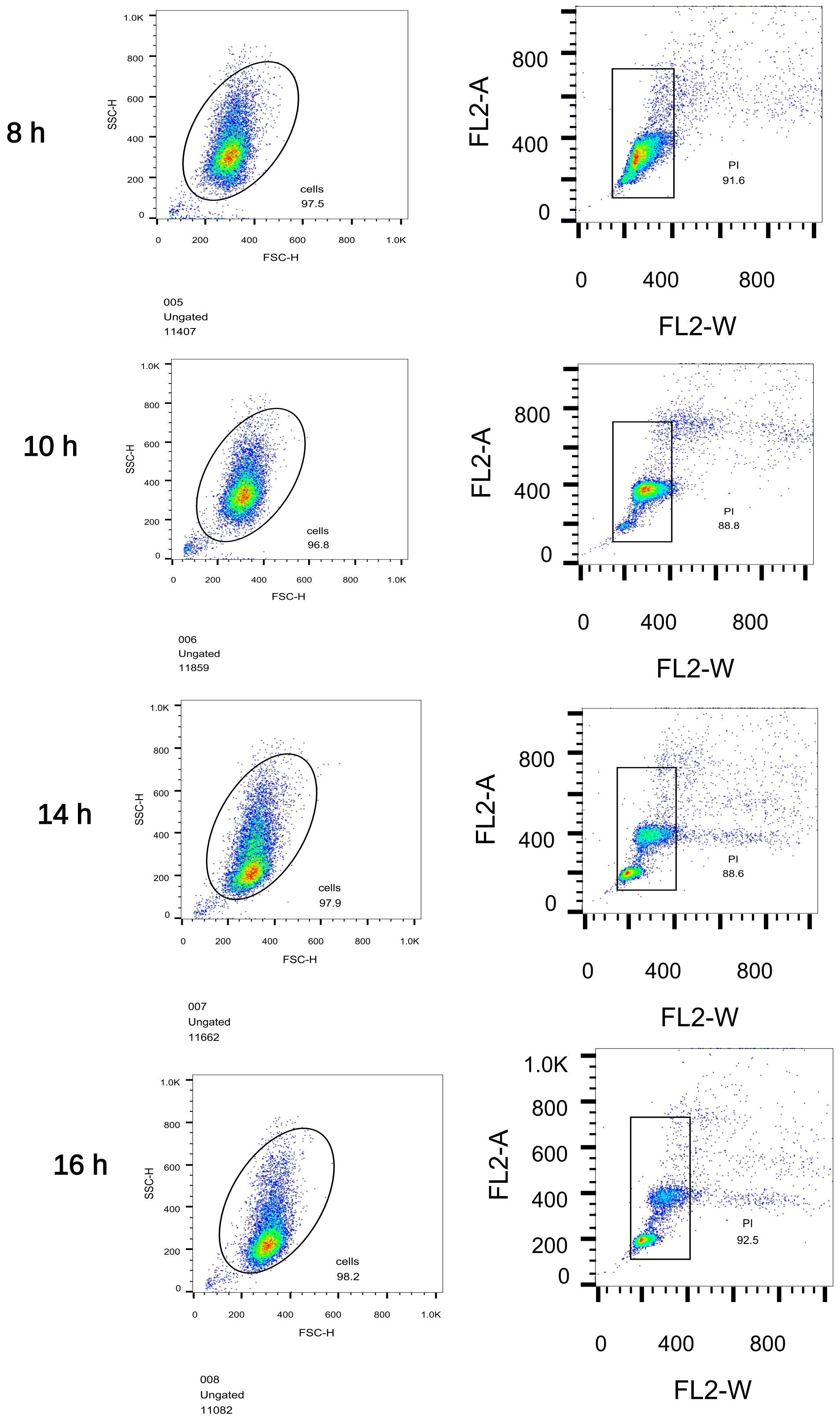

FACS information for Fig. 6 and Supplementary Fig. 2

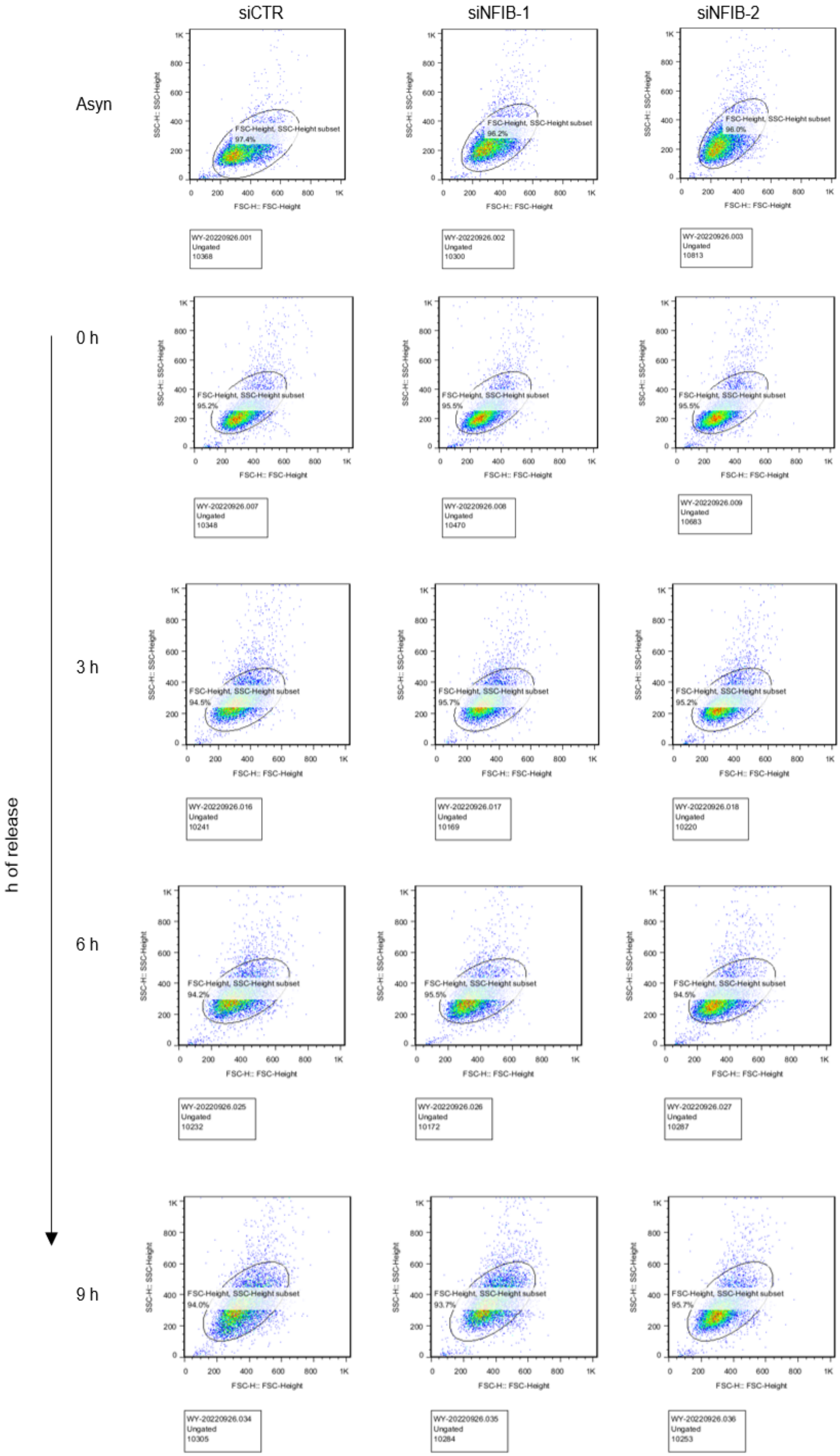

Supplement: Supplementary file 1 — Supplementary Information [file 41467_2023_40846_MOESM1_ESM.pdf]
