# Supplementary material for: NFIB facilitates replication licensing by acting as a genome organizer
Source: Nat Commun. 2023 Aug 21;14:5076. doi: 10.1038/s41467-023-40846-1 (PMC10442334; doi:10.1038/s41467-023-40846-1)
Supplement: Supplementary file 2 — Reporting Summary [file 41467_2023_40846_MOESM2_ESM.pdf]

nature portfolio

Corresponding author(s): Yongfeng Shang

Last updated by author(s): Jul 26, 2023

Reporting Summary

Nature Portfolio wishes to improve the reproducibility of the work that we publish. This form provides structure for consistency and transparency in reporting. For further information on Nature Portfolio policies, see our Editorial Policies and the Editorial Policy Checklist.

Statistics

For all statistical analyses, confirm that the following items are present in the figure legend, table legend, main text, or Methods section.

n/a	Confirmed	
☐	☒	The exact sample size (*n*) for each experimental group/condition, given as a discrete number and unit of measurement
☐	☒	A statement on whether measurements were taken from distinct samples or whether the same sample was measured repeatedly
☐	☒	The statistical test(s) used AND whether they are one- or two-sided<br>*Only common tests should be described solely by name; describe more complex techniques in the Methods section.*
☒	☐	A description of all covariates tested
☐	☒	A description of any assumptions or corrections, such as tests of normality and adjustment for multiple comparisons
☐	☒	A full description of the statistical parameters including central tendency (e.g. means) or other basic estimates (e.g. regression coefficient) AND variation (e.g. standard deviation) or associated estimates of uncertainty (e.g. confidence intervals)
☐	☒	For null hypothesis testing, the test statistic (e.g. *F*, *t*, *r*) with confidence intervals, effect sizes, degrees of freedom and *P* value noted<br>*Give P values as exact values whenever suitable.*
☒	☐	For Bayesian analysis, information on the choice of priors and Markov chain Monte Carlo settings
☒	☐	For hierarchical and complex designs, identification of the appropriate level for tests and full reporting of outcomes
☐	☒	Estimates of effect sizes (e.g. Cohen's *d*, Pearson's *r*), indicating how they were calculated

Our web collection on statistics for biologists contains articles on many of the points above.

Software and code

Policy information about availability of computer code

Data collection	Zeiss ZEN (black), Illumina Hiseq2000/Nova seq, BD FACS Diva 8.0.1, BD Calibur 2, ImageJ
Data analysis	Zeiss ZEN v2.3 (blue), GraphPad Prism 6, FlowJo v.10, Bowtie v2.2.5, bedtools v2.17.0, R v3.4.3, MACS2 v1.4.1, python v2.7.6, tophat v2.2.1, cufflinks v2.2.1, samtools v1.2.1, deeptools v2.3.5, IGV v2.3, PeakSeq v1.3, FASTX-Tools v 0.0.13

For manuscripts utilizing custom algorithms or software that are central to the research but not yet described in published literature, software must be made available to editors and reviewers. We strongly encourage code deposition in a community repository (e.g. GitHub). See the Nature Portfolio guidelines for submitting code & software for further information.

Data

Policy information about availability of data

All manuscripts must include a data availability statement. This statement should provide the following information, where applicable:

- Accession codes, unique identifiers, or web links for publicly available datasets
- A description of any restrictions on data availability
- For clinical datasets or third party data, please ensure that the statement adheres to our policy

The raw high-throughput sequencing data generated in this study have been deposited in the NCBI Gene Expression Omnibus (GEO) database under accession code GSE201867 (https://www.ncbi.nlm.nih.gov/geo/query/acc.cgi?acc=GSE201867). The reference dataset used in the study is a human reference genome (UCSC

GRCh37, hg38). The source data for Figs 1a-g, 2a, 2b, 2e, 4b-4e, 5a-5f, 5h, 6a, 6c-6i, 7d, 7e, 8a-8d and Supplementary Figs 1a, 2b-2c, 3a-3c, 5a, 5b are provided in Source Data file.

Research involving human participants, their data, or biological material

Policy information about studies with human participants or human data. See also policy information about sex, gender (identity/presentation), and sexual orientation and race, ethnicity and racism.

Reporting on sex and gender	This information has not been collected as no human research participants in our study.
Reporting on race, ethnicity, or other socially relevant groupings	See above.
Population characteristics	See above.
Recruitment	See above.
Ethics oversight	See above.

Note that full information on the approval of the study protocol must also be provided in the manuscript.

Field-specific reporting

Please select the one below that is the best fit for your research. If you are not sure, read the appropriate sections before making your selection.

☒ Life sciences ☐ Behavioural & social sciences ☐ Ecological, evolutionary & environmental sciences

For a reference copy of the document with all sections, see nature.com/documents/nr-reporting-summary-flat.pdf

Life sciences study design

All studies must disclose on these points even when the disclosure is negative.

Sample size	To do the statistical test, at least 3 biological replicates were included (unless otherwise stated) based on previously published work and preliminary studies as standard for this field of research.
Data exclusions	No data were excluded from the analyses.
Replication	All experiments were reliably reproduced. Each experiment was performed independently at least two times, but usually many more times.
Randomization	Cells were allocated at random to experimental groups as stated in the Methods.
Blinding	Blinding was not performed due to the unambiguous nature of measurements and systematic analyses used in these experiments.

Reporting for specific materials, systems and methods

We require information from authors about some types of materials, experimental systems and methods used in many studies. Here, indicate whether each material, system or method listed is relevant to your study. If you are not sure if a list item applies to your research, read the appropriate section before selecting a response.

Materials & experimental systems

n/a	Involved in the study	
☐	☒	Antibodies
☐	☒	Eukaryotic cell lines
☒	☐	Palaeontology and archaeology
☒	☐	Animals and other organisms
☒	☐	Clinical data
☒	☐	Dual use research of concern
☒	☐	Plants

Methods

n/a	Involved in the study	
☐	☒	ChIP-seq
☐	☒	Flow cytometry
☒	☐	MRI-based neuroimaging

Antibodies

Antibodies used	Antibodies used and the sources are as follows: NFIB (ab186738, 1:1000 for WB), ORC1 (ab85830, 1:1000 for WB), CDC6 (ab188423,

Antibodies used	1:1000 for WB), CDT1 (ab70829, 1:1000 for WB), MCM5 (ab76023, 1:1000 for WB), MCM6 (ab201683, 1:1000 for WB), MCM7 (ab52489, 1:1000 for WB), PCNA (ab29, 1:2000 for WB), H3 (ab1791, 1:2000 for WB) and BrdU (anti-CldU, ab6326) from Abcam; MCM2 (3619, 1:1000 for WB) from Cell Signaling Technology; ?RC2 (sc-32734, 1:500 for WB), Geminin (sc-74456, 1:1000 for WB), POLD1 (sc-17776, 1:1000 for WB) and ?-actin (sc-47778, 1:2000 for WB) from Santa Cruz Biotechnology; MCM4 (13043-1-AP, 1:1000 for WB), Cyclin E1 (11554-1-AP, 1:1000 for WB) and MCM3 (A1060, 1:1000 for WB) from Abclonal; NFIB (A303-566A, for CUT&Tag) and ORC1 (A301-892A, for CUT&Tag) from Bethyl Laboratories; c-myc-Tag (B1022, 1:2000 for WB), HA-Tag (B1021, 1:2000 for WB) and GAPDH (B1034, 1:2000 for WB) from Biodragon. FITC or TRITC-conjugated secondary antibodies (ZF-0311, ZF-0312, ZF-0316, ZF-0313, 1:100 for IF) from ZSGB-BIO.
Validation	All antibodies used are commonly used in the field and have been validated in previous publications/by the manufacturer.<br>References and manufacturer validations can be found here:<br>Anti-NFIB (Abcam ab186738):https://www.abcam.cn/nfib--nf1b2-antibody-epr14122-ab186738.html<br>Anti-ORC1 (Abcam ab85830):https://www.abcam.cn/orc1-antibody-ab85830.html<br>Anti-CDC6 (Abcam ab188423):https://www.abcam.cn/cdc6-antibody-n-terminal-ab188423.html<br>Anti-CDT1 (Abcam ab70829):https://www.abcam.cn/cdt1dup-antibody-ab70829.html<br>Anti-MCM5 (Abcam ab76023):https://www.abcam.cn/mcm5-antibody-ep2682y-ab76023.html<br>Anti-MCM6 (Abcam ab201683):https://www.abcam.cn/mcm6-antibody-epr17686-ab201683.html<br>Anti-MCM7 (Abcam ab52489):https://www.abcam.cn/mcm7prl-antibody-ep1974y-ab52489.html<br>Anti-PCNA (Abcam ab29):https://www.abcam.cn/pcna-antibody-pc10-ab29.html<br>Anti-H3 (Abcam ab1791):https://www.abcam.cn/histone-h3-antibody-nuclear-marker-and-chip-grade-ab1791.html<br>Anti-BrdU (Abcam, ab6326):https://www.abcam.cn/brdu-antibody-bu175-icr1-proliferation-marker-ab6326.html<br>Anti-MCM2 (CST 3619):https://www.cellsignal.cn/products/primary-antibodies/mcm2-d7g11-xp-rabbit-mab/3619;jsessionid=oaydytoulewid8wyj6uust38fgzaqt44acwkpmfo.prod_store02?N=4294956287&Ntt=MCM2+%25283619%2529&_requestid=5072109&fromPage=plp&site-search-type=Products<br>Anti-ORC2 (Santa Cruz Biotechnology sc-32734):https://www.scbt.com/p/orc2-antibody-3g6?requestFrom=search<br>Anti-Geminin (Santa Cruz Biotechnology sc-74456):https://www.scbt.com/p/geminin-antibody-f-7?requestFrom=search<br>Anti-POLD1 (Santa Cruz Biotechnology sc-17776):https://www.scbt.com/p/dna-pol-delta-cat-antibody-a-9?requestFrom=search<br>Anti-β-actin (Santa Cruz Biotechnology sc-47778):https://www.scbt.com/p/beta-actin-antibody-c4?requestFrom=search<br>Anti-MCM4 (Proteintech 13043-1-AP):https://www.ptgcn.com/products/MCM4-Antibody-13043-1-AP.htm<br>Anti-Cyclin E1 (Proteintech 11554-1-AP):https://www.ptgcn.com/products/CCNE1-Antibody-11554-1-AP.htm<br>Anti-MCM3 (Abclonal A1060):https://abclonal.com.cn/catalog/A1060<br>Anti-NFIB (Bethyl Laboratories A303-566A, for CUT&Tag):https://www.thermofisher.cn/cn/zh/antibody/product/NFIB-Antibody-Polyclonal/A303-566A<br>Anti-ORC1 (Bethyl Laboratories A301-892A, for CUT&Tag):https://www.thermofisher.cn/cn/zh/antibody/product/ORC1-Antibody-Polyclonal/A301-892A<br>Anti-c-myc-Tag (Biodragon B1022):https://www.biodragon.cn/plus/view.php?aid=74615<br>Anti-HA-Tag (Biodragon B1021):https://www.biodragon.cn/plus/view.php?aid=74629<br>Anti-GAPDH (Biodragon B1034):https://www.biodragon.cn/nckt/73662.html

Eukaryotic cell lines

Policy information about cell lines and Sex and Gender in Research

Cell line source(s)	Cell lines (U2OS, HEK293T, MCF-10A, BT-474, ZR-75-1, SF9) used were obtained from the American Type Culture Collection (ATCC).
Authentication	Identity of cell line was frequently checked and authenticated by the morphological features and STR analysis.
Mycoplasma contamination	The cell lines were tested negative for mycoplasma contamination.
Commonly misidentified lines<br>(See ICLAC register)	No commonly misidentified cell lines were used.

Plants

Seed stocks	This information has not been collected as no plant research participants in our study.
Novel plant genotypes	See above.
Authentication	See above.

ChIP-seq

Data deposition

☒ Confirm that both raw and final processed data have been deposited in a public database such as GEO.

☒ Confirm that you have deposited or provided access to graph files (e.g. BED files) for the called peaks.

Data access links<br>*May remain private before publication.*	https://www.ncbi.nlm.nih.gov/geo/query/acc.cgi?acc=GSE201867
Files in database submission	GSM6077281 NC-NFIB-Cuttag

Files in database submission	GSM6077282 NC-ORC1-Cuttag<br>GSM6077283 shNFIB-NFIB-Cuttag<br>GSM6077284 shNFIB-ORC1-Cuttag<br>GSM6077285 Microc_G1_NC_1<br>GSM6077286 Microc_G1_NC_2<br>GSM6077287 Microc_S_NC_1<br>GSM6077288 Microc_S_NC_2<br>GSM6077289 Microc_G1_sh_1<br>GSM6077290 Microc_G1_sh_2<br>GSM6077291 Microc_S_sh_1<br>GSM6077292 Microc_S_sh_2<br>GSM6077293 NC-NS<br>GSM6077294 NC-NS-RNase<br>GSM6077295 shNFIB-NS<br>GSM6077296 shNFIB-NS-RNase<br>GSM6077297 5-fluoracil-WGS-1<br>GSM6077298 5-fluoracil-WGS-2<br>GSM6077299 NFIB-WGS-30d-1<br>GSM6077300 NFIB-WGS-30d-2<br>GSM6077301 NFIB-WGS-30d-3<br>GSM6077302 NFIB-WGS-30d-4<br>GSM6077303 NFIB-WGS-75d-1<br>GSM6077304 NFIB-WGS-75d-2<br>GSM6077305 Oxaliplatin-WGS-1<br>GSM6077306 Oxaliplatin-WGS-2<br>GSM6077307 Vector-WGS-30d-1<br>GSM6077308 Vector-WGS-30d-2<br>GSM6077309 Vector-WGS-30d-3<br>GSM6077310 Vector-WGS-30d-4<br>GSM6077311 Vector-WGS-30d-5<br>GSM6077312 Vector-WGS-30d-6<br>GSM6077313 Vector-WGS-75d-1<br>GSM6077314 Vector-WGS-75d-2<br>GSM6953328 ATAC_NC-G1-1<br>GSM6953329 ATAC_NC-G1-2<br>GSM6953330 ATAC_NC-S-1<br>GSM6953331 ATAC_NC-S-2<br>GSM6953332 ATAC_si-G1-1<br>GSM6953333 ATAC_si-G1-2<br>GSM6953334 ATAC_si-S-1<br>GSM6953335 ATAC_si-S-2<br>GSM6953336 CUT&Tag NC-K4-G1<br>GSM6953337 CUT&Tag NC-K4-S<br>GSM6953338 CUT&Tag si-K4-G1<br>GSM6953339 CUT&Tag si-K4-S<br>GSM6953376 CUT&Tag NC-G1-K9<br>GSM6953377 CUT&Tag NC-S-K9<br>GSM6953378 CUT&Tag si-G1-K9<br>GSM6953379 CUT&Tag si-S-K9<br>GSM6953380 Repli NC-1-1R<br>GSM6953381 Repli NC-1-4R<br>GSM6953382 Repli NC-1-6R<br>GSM6953383 Repli NC-2-1R<br>GSM6953384 Repli NC-2-4R<br>GSM6953385 Repli NC-2-6R<br>GSM6953386 Repli si-1-1R<br>GSM6953387 Repli si-1-4R<br>GSM6953388 Repli si-1-6R<br>GSM6953389 Repli si-2-1R<br>GSM6953390 Repli si-2-4R<br>GSM6953391 Repli si-2-6R
Genome browser session<br>(e.g. UCSC)	Not used

Methodology

Replicates	ATAC-seq and Repli-seq experiments were performed in duplicates.
Sequencing depth	Sample#Reads#Mapping#Type<br>NC-NFIB-Cuttag.R1.fastq.gz 40362280 40095364 PE<br>NC-NFIB-Cuttag.R2.fastq.gz 40362280 40095364 PE<br>NC-ORC1-Cuttag.R1.fastq.gz 131659800 126105582 PE<br>NC-ORC1-Cuttag.R2.fastq.gz 131659800 126105582 PE<br>shNFIB-NFIB-Cuttag.R1.fastq.gz 48868839 48309458 PE<br>shNFIB-NFIB-Cuttag.R2.fastq.gz 48868839 48309458 PE

	shNFIB-ORC1-Cuttag.R1.fastq.gz 180633825 173215276 PE<br>shNFIB-ORC1-Cuttag.R2.fastq.gz 180633825 173215276 PE
Antibodies	NFIB (A303-566A, for CUT&Tag) and ORC1 (A301-892A, for CUT&Tag) from Bethyl Laboratories.
Peak calling parameters	Reads were uniquely mapped to genome using bowtie2. Peaks were called using MACS2 (FDR below 0.05)
Data quality	Read with high quality were retained using Fastx_toolkit, unique reads were used for peak calling with FDR below 0.5%
Software	Bowtie v2.2.5, bedtools v2.17.0, R v3.4.3, MACS2 v1.4.1, python v2.7.6, tophat v2.2.1, cufflinks v2.2.1, samtools v1.2.1, DESeq2, deeptools v2.3.5, IGV v2.3, PeakSeq v1.3, FASTX-Tools v 0.0.13,

Flow Cytometry

Plots

Confirm that:

☒ The axis labels state the marker and fluorochrome used (e.g. CD4-FITC).

☒ The axis scales are clearly visible. Include numbers along axes only for bottom left plot of group (a 'group' is an analysis of identical markers).

☒ All plots are contour plots with outliers or pseudocolor plots.

☒ A numerical value for number of cells or percentage (with statistics) is provided.

Methodology

Sample preparation	For cell cycle analysis:<br>1. Cells were collected, washed and resuspended with cold PBS, and fixed in chilled ethanol overnight. Cells were then washed and resuspended in PBS with 120 µg/ml propidium iodide (PI) and 10 µg/ml RNase A for 30 min.<br>2. Cells were pulse-labeled with 10 µM EdU for 1 h. After harvesting and washing with 1% BSA in PBS, cells were fixed in Click-iT® fixative for 15 min at RT. Cells were dislodged in 1 × Click-iT® saponin-based permeabilization and wash reagent at RT for 15 min. Click-iT® reactions were performed with Alexa Fluor® 647 azide at RT for 30 min. After digesting RNAs with RNase A and staining cells with propidium iodide, flow cytometry was performed in a FACS Calibur2 (BD) and data analyzed with FlowJo v.10.
Instrument	BD Calibur 2
Software	BD FACS Diva 8.0.1 software was used for data collection and FlowJo v.10 was used for data analysis.
Cell population abundance	For the cell cycle, there are ~65% of G1/G0 phase cell, ~25% of S phase cell and ~10% of M phase cell in asynchronized cells, ~85% of G1/G0 phase cell, ~7.5% of S phase cell and ~7.5% of M phase cell in synchronized G1/S phase cells.
Gating strategy	For cell cycle analysis:<br>Single alive U2OS were gated with PI.<br>Single alive U2OS, MCF-10A , BT-474 cells were gated with Brdu and PI. Brdu low and PI low cells were resident G1/G0 phase cells; Brdu high and PI middle cells were resident S phase cell cells; Brdu low and PI high cells were resident M phase cell cells.

☒ Tick this box to confirm that a figure exemplifying the gating strategy is provided in the Supplementary Information.
